# Effect of a Semisolid Formulation of *Linum usitatissimum* L. (Linseed) Oil on the Repair of Skin Wounds

**DOI:** 10.1155/2012/270752

**Published:** 2011-06-14

**Authors:** Eryvelton de Souza Franco, Camilla Maria Ferreira de Aquino, Paloma Lys de Medeiros, Liriane Baratella Evêncio, Alexandre José da Silva Góes, Maria Bernadete de Souza Maia

**Affiliations:** ^1^Department of Physiology and Pharmacology, Federal University of Pernambuco, 50670-901 Recife, PE, Brazil; ^2^Department of Histology and Embryology, Federal University of Pernambuco, 50670-901 Recife, PE, Brazil; ^3^Department of Antibiotics, Federal University of Pernambuco, 50670-901 Recife, PE, Brazil; ^4^Laboratory of Pharmacology of Bioactive Products, Department of Physiology and Pharmacology, Federal University of Pernambuco, Avenida Professor Moraes Rêgo, S/N, Cidade Universitária, 50670-901 Recife, PE, Brazil

## Abstract

The purpose of this study was to investigate the effects of a semisolid formulation of linseed oil, SSFLO (1%, 5%, or 10%) or *in natura* linseed oil on skin wounds of rats. We used wound models, incisional and excisional, to evaluate, respectively, the contraction/reepithelialization of the wound and resistance to mechanical traction. The groups (*n* = 6) treated with SSFLO (1% or 5%) began the process of reepithelialization, to a significant extent (*P* < .05), on the sixth day, when compared to the petroleum jelly control group. On 14th day for the groups treated with SSFLO (1% or 5%), 100% reepithelialization was found, while in the petroleum jelly control group, this was only 33.33%. Our study showed that topical administration of SSFLO (1% or 5%) in excisional wounds allowed reepithelialization in 100% of treated animals. Therefore, a therapeutic potential of linseed oil, when used at low concentrations in the solid pharmaceutical formulations, is suggested for the process of dermal repair.

## 1. Introduction

*Linum usitatissimum* L. is a herb belonging to the Linaceae family, popularly known as flax or linseed, which is native to Europe, Asia, and the Mediterranean region. The seeds of flax are used as the source material for the production of oil and meal, which is rich in fiber, protein, and fat [1]. Its oil is arguably one of the richest in polyunsaturated fatty acids (PUFA) and essential fatty acids (EFA), with 56.6% linolenic acid [*ω*-3 (C18: 3 cis 9,12,15)] and 13.2% linoleic acid [*ω*-6 (C18: 2 cis 9,12)], in addition to 17.8% monounsaturated fatty acid (MUFA) oleic acid [*ω*-9 (C18: 1 cis 9)] [2].

Linseed oil is present in the Brazilian national pharmacopoeia in the form of calcareous liniment oil, which is indicated for use in cases of pruritic dermatoses and burns [3]. According to Chinese traditional medicine and ayurvedic medicine, linseed oil is indicated for the treatment of wounds and as a moisturizer and dermal antioxidant, among other uses [4]. The pharmacological properties of linseed oil are attributed to the presence of PUFA and MUFA in its composition, which act stimulating production of growth factors, fibroplasia, and neovascularization [5, 6]. 

Previouly authors reported that linoleic acid (*ω*-6) exhibits proinflammatory characteristics, whereas linolenic acid (*ω*-3) and oleic acid (*ω*-9) have an anti-inflammatory effect [7]. In a study evaluating the effect of topical administration of purified linolenic, linoleic and oleic acid, on the process of wound healing in rats, Cardoso et al. [8] observed that in the groups treated topically with MUFA oleic or PUFA linoleic showed significant reduction in wound area when compared with the group treated with PUFA linolenic acid, suggesting a favorable effect of MUFA oleic and PUFA linoleic acids on the process of tissue repair, specifically as proinflammatory agents during the inflammatory phase of the healing process [8, 9]. It is noteworthy that linoleic acid (*ω*-6) is the direct precursor of arachidonic acid, which is part of the membrane phospholipids and which prevents transepidermal water loss, provides elasticity, skin integrity and fluidity [10], and serves as a substrate for the synthesis of eicosanoids such as prostaglandins, leukotrienes and thromboxanes [11]. These, in turn, modulate immunological and inflammatory responses by altering leukocyte functions and accelerating the process of tissue granulation [12].

Taking into consideration the scientific background regarding the chemical composition of linseed oil as a major source of *ω*-3 and *ω*-6 PUFA, we undertook this study to explore two cases of healing activity using a semisolid formulation of linseed oil in experimental models of skin wounds.

## 2. Methods

### 2.1. Preparation of the Pharmaceutical Formulation

The semisolid formulation of linseed oil (SSFLO) was composed of commercial linseed oil (Cisbra vegetable oils LTDA, Ijuí-Rio Grande do Sul, Brazil) (*L. usitatissimum*), extracted by cold-pressing the seeds, and a vehicle (petroleum jelly), in sufficient amount to obtain 100 g of SSFLO (1%, 5%, and 10%). The procedure was handled within the standards and quality control for medicines from the Medical Technology Laboratory, Pharmaceutical Faculty of the Federal University of Pernambuco. As a negative control the formulation vehicle (petroleum jelly) was used and as a positive control, an oil emulsion based on sunflower oil rich in EFA (Dersani) (ESO).

### 2.2. Biological Models

A total of 72 Wistar rats (males and females (200–300 g)), from the vivarium of the Department of Pharmacology and Physiology of the Federal University of Pernambuco were used. The animals were randomly divided into groups (*n* = 6 animals) and kept individually in metabolic cages, with food and water *ad libitum*.

### 2.3. Bioethical Considerations

The study was approved by the ethics committee for animal experimentation (number 23076007695/2009-19) of the Federal University of Pernambuco, Recife, Brazil.

### 2.4. Excisional Wound Model

**Figure 1:**
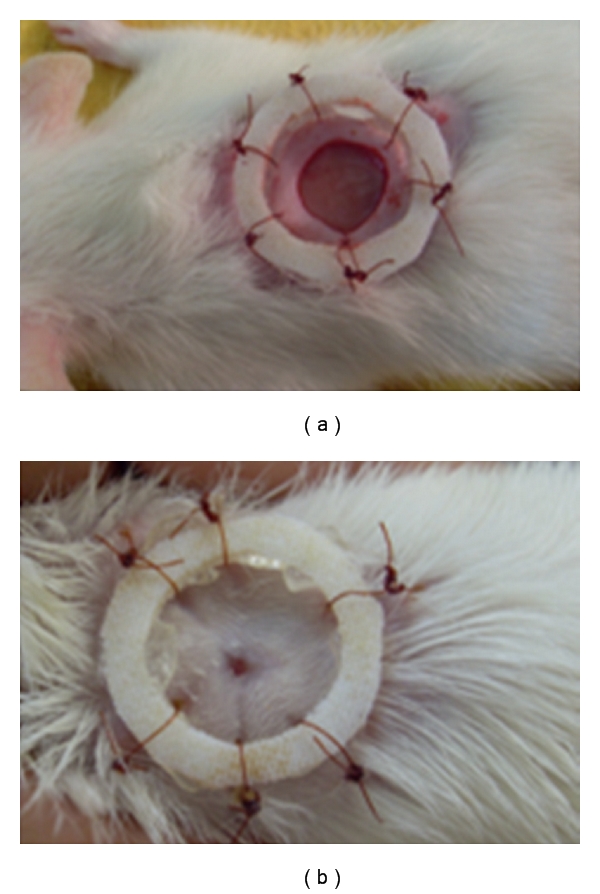
(a) Excision wound on day 1 (control group animal). (b) Excision wound on day 14 (control group animal).

Wistar rats (*n* = 36) were weighed and anesthetized with xylazine (3 mg kg^−1^) and ketamine (10 mg kg^−1^ i.m.) [13]. Subsequently the animals were placed in a prone position and underwent manual trichotomy and antisepsis with 0.1% iodine alcohol along the dorsal midline of the cervical region of each animal. The incision area was marked using a surgical pen and metal mold castings (±78.5 mm^2^). To remove the skin, subcutaneous tissue, fascia, and fleshy panniculus, straight iris scissors and the Adson forceps were used. After the incision, a containment ring made of nontoxic and hypoallergenic silicone was sutured into place, using six simple, isolated stitches with 4.0 nylon monofilament, arranged symmetrically [14], so that the surgical wound would remain in the center (Figures 1(a) and 1(b)).

After surgery, the animals were divided randomly into six groups (*n* = 6 animals/group) and treated over 14 consecutive days with 1% SSFLO, 5% SSFLO, 10% SSFLO, *in natura* linseed oil, ESO (positive control) or petroleum jelly (negative control). The topical application of each product (95 mg) was done using a metal spatula. On the seventh day, the scabs of the wounds on each animal were removed to allow the evaluation of the tissue that was below them and the true value of the remaining wound area.

### 2.5. Macroscopic Analysis of Wound Closure

To assess the contraction of wounds treated with the different products, the wounds were measured daily using a digital caliper according to the equation formulated by Prata et al. [15], *A* = *π* · *R* · *r*, where *A* represents the area (mm²), “*R*” the larger radius, and “*r*” the smaller radius. The assessments were made by the same examiner, with the animals under physical restraint. The wounds were also evaluated for the presence of exudate, scab formation, early reepithelialization and the presence of fibrin (slough).

### 2.6. Microscopic Morphometric Analysis

At the end of the experiment (14th day of treatment), the animals were euthanized in a CO_2_ chamber and then placed on an operating table for the collection of material for the morphometric analysis of the skin lesions. All the samples obtained were fixed in 10% buffered formalin for a minimum period of 24 hours, and subsequently dehydrated in ethanol and cleared in xylene. Next, the samples were processed for inclusion in paraffin by routine methods and cut using a Minot-type microtome set to 4 *μ*m. The material obtained was placed on slides previously coated with Mayer's albumin.

After drying, the sections were stained with hematoxylin-eosin [16] for observations of general tissue morphology and Masson's Trichrome [17] to analyze the organization of collagen fibrils in the dermis. The slides were examined under light microscope, five images per field (0.0018 mm^2^ area) were captured with a digital camera (total magnification 400x) attached to the microscope (Olympus BX-49 light microscope and camera). The images were stored and subjected to counting of inflammatory cells, fibroblast cells, number of blood vessels, and evaluation of collagen density of all lesions with the aid of digital marking [18, 19]. 

### 2.7. Incisional Wound Model

Wistar rats (*n* = 36) were weighed and intramuscularly anesthetized with xylazine (3 mg kg^−1^) and ketamine (10 mg kg^−1^) [13]. The animals were then placed in a prone position and subjected to manual trichotomy and antisepsis with 0.1% iodine alcohol in the region of the dorsal midline. Later, an incision was made using a number 15 scalpel blade, parallel to the spine three centimeters in length. The preestablished skin flap was comprised of the skin, subcutaneous tissue, and superficial fascia to the point of the fleshy panniculus. The site was dissected with blunt dissection to offset the adjacent muscle-aponeurotic plane then repositioned and sutured with two simple stitches using 4.0 nylon monofilament [20]. After surgery, the animals were divided randomly into six groups (*n* = 6 animals/group) and treated over 10 consecutive days with 1% SSFLO, 5% SSFLO, 10% SSFLO, *in natura* linseed oil, ESO (positive control), or petroleum jelly (negative control). The sutures were removed on the eighth day, and the wounds continued to be treated until the evaluation of skin resistance on the tenth day. For this assessment, the animals were euthanized in a CO_2_ chamber and the skin of the wound site was removed and evaluated for resistance using a universal mechanical test, with manually adjustable pressure application and a computerized system of data acquisition [21].

### 2.8. Statistical Analysis

All variables (wound area, inflammatory cells, fibroblast cells, number of blood vessels, collagen density, and tensile strength of the skin and presence of scabs, the presence of exudate and the presence of tissue reepithelialization) were expressed as mean ± standard deviation and subjected, respectively, to one-way ANOVA (quantification of the variables on the last day of treatment) or two-way ANOVA (presence of the variables during each day of treatment (quantity × time) followed by *Bonferroni's* multiple test comparing the SSFLO treatment groups (1%, 5%, or 10%) or linseed oil to the petroleum jelly and ESO controls, considering significant values (*P* < .05). Data were analyzed using Graph Pad Instant version 5.0 (GraphPad Software, San Diego, Calif/USA). 

## 3. Results

### 3.1. Development of Excisional Wounds

**Figure 2:**
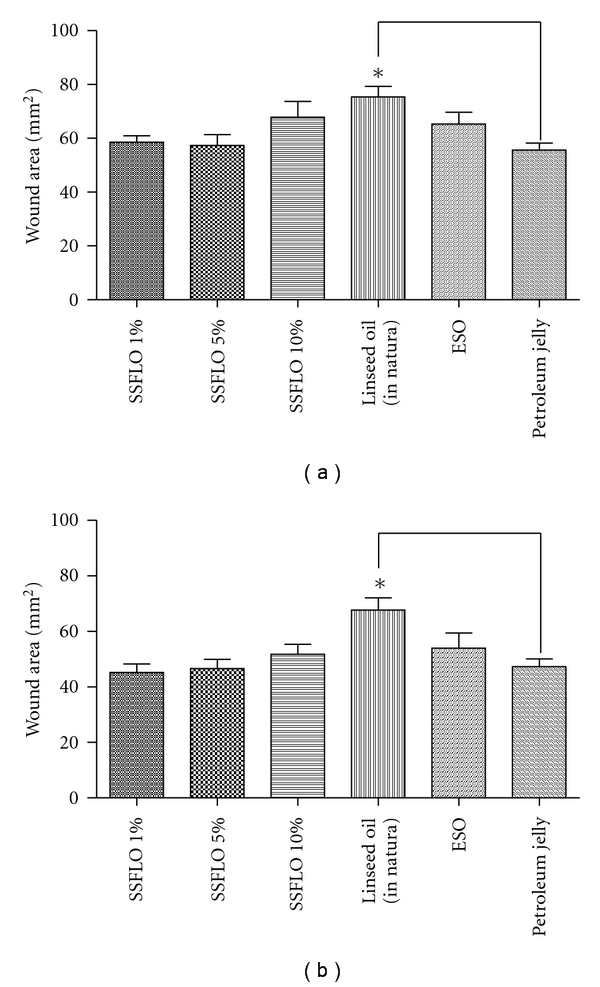
Effect of treatment with SSFLO (1%, 5% or 10%) compared to control groups, ESO and petroleum jelly reducing the area of wounds, excision model. (a) After four days of treatment, (b) after five days of treatment. *(*P* < .05).

To assess whether treatment with SSFLO (1%, 5% or 10%) or *in natura* linseed oil influenced the closing time of second-intention wounds, compared to the ESO or petroleum jelly control groups, the wounds of each animal were measured daily. We observed a significant reduction (*P* < .05) of wound contraction in the group treated with linseed oil when compared to the petroleum jelly control on the fourth and fifth days of treatment (Figures 2(a) and 2(b)). However, from the sixth day of treatment, tissue repair occurred uniformly in all groups, with no observed significant difference in wound contraction.

There was a significant decrease (*P* < .05) in the presence of scabs in the group treated with 1% SSFLO on the second day (one animal—16.67%) when compared to the ESO control group (five animals—83.33%). For the group treated with *in natura* linseed oil, we observed a significant increase in the presence of scabs (*P* < .05) on the third day (six animals—100%), when compared to the petroleum jelly control (two animals—33.33%). On the other days, there was no significant difference regarding the presence or absence of scabs, when comparing the treatment groups to the controls.

In all groups a serous exudate was observed in the early hours after surgery. However, during the fourteen-day trial there was no significant difference regarding the presence of exudate in the treated groups compared to the controls.

The removal of the scab was necessary on the seventh day, to allow for better contact of the SSFLO (1%, 5% and 10%) or *in natura* linseed oil and visualization of the wound bed [22]. With this removal, it was possible to assess, macroscopically, the type of tissue present. A significant (*P* < .05) increase in the presence of fibrin (slough) was found in the groups treated with SSFLO 1% (three animals—50%) in SSFLO 5% (one animal—16.67%) or 10% (two animals—33.33%) compared with the ESO control group, which did not show formation of slough in the wound bed. On the same day, a significant (*P* < .05) presence of slough in the group treated with SSFLO 1% (three animals—50%) compared to the petroleum jelly control (one animal—16.67%).

A significant (*P* < .05) increase in tissue reepithelialization was seen from the sixth day, in groups treated with SSFLO 1% (four animals—66.67%) or 5% (four animals—66.67%) compared to the ESO control group (two animals—33.33%). This same variable was significantly (*P* < .05) lower on the sixth day, in groups treated with 10% SSFLO (three animals—50%) or *in natura* linseed oil (three animals—50%) compared to the petroleum jelly control group (five animals—83.33%).

Microscopic morphometric analysis: To determine whether treatment with SSFLO (1%, 5% or 10%) or *in natura *linseed oil influenced the second intention repair process of wounds in terms of reepithelialization, presence of dermal papillae, number of blood vessels, number of fibroblast cells, and collagen density, we evaluated the histological morphology of the dermal region after 14 days of treatment and compared it to the ESO and petroleum jelly control groups.

**Figure 3:**
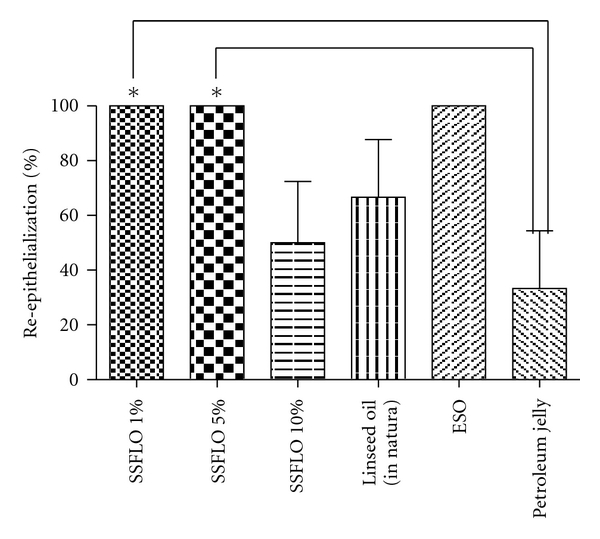
Effect of treatment with SSFLO (1%, 5%, or 10%) compared to control groups, petroleum jelly and ESO on the reepithelialization (%) of excisional wound model, after 14 days. *(*P* < .05).

It was observed that 100% of animals in groups treated with SSFLO (1% or 5%) showed complete reepithelialization at the end of the experiment, this result was significant (*P* < .05) when compared with the petroleum jelly control group (Figure 3).

**Figure 4:**
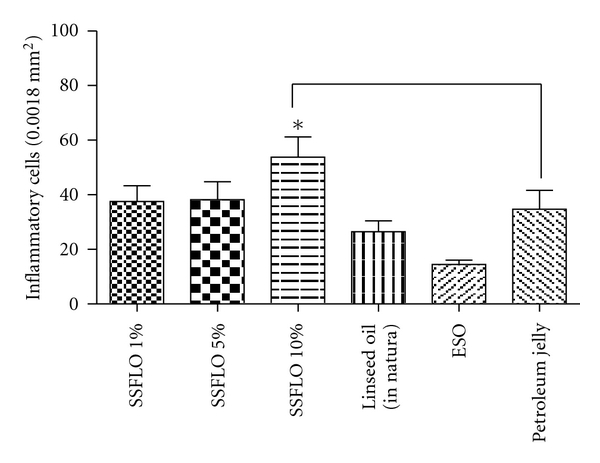
Effect of treatment with SSFLO (1%, 5%, or 10%) compared to the control groups petroleum jelly and ESO on the amount of inflammatory cells in scar tissue, excisional wound model, after 14 days. *(*P* < .05).

In the morphometric evaluation a significant increase (*P* < .05) in the number of inflammatory cells in the group treated with 10% SSFLO was observed compared to the ESO control group (Figure 4). Among the remaining variables, there were no significant differences observed.

### 3.2. Tensile Strength Study of Cutaneous Wounds

Tensile strength evaluation seems highly relevant, since it has been used in several studies of healing, and is a physical parameter that, in connection with the histological evaluation, allows us to deduce the quality of the healing process, as well as the resistance of the skin to the tension suffered. There was no significant difference in tensile strength among the different experimental groups.

## 4. Discussion

In the present study, standardized surgical wounds were prepared, which were submitted to the repair process in the first intention (incisional model) and second intention (excisional model) for ten or fourteen days of treatment, respectively, with SSFLO (1%, 5%, or 10%) or *in natura* linseed oil. Howel and Maquart [23] defined as cure or wound repair the healing process, which may occur in the first intention, where there is immediate union of the edges or, in the second intention, where the edges are separated and there is need to form scar tissue. The use of models allows the evaluation of the effect of the products concerned, on the mechanism of action wound contraction, through possible stimulation of tissue repair and resistance to the pull of the skin, which would be associated with the stimulation of collagen synthesis [4, 24].

We observed a significant increase in the area of the wounds treated with *in natura* linseed oil on the fourth and fifth days, when compared to the petroleum jelly control. This result can be explained by the occlusive and moisturizing action of petroleum jelly that, through this mechanism, prevents tissue from drying and prevents ischemia of deeper tissues, preventing the increase of the lesion [25]. This result does not occur with *in natura* linseed oil, as it occurs in liquid form and is rapidly absorbed, allowing the wound bed to become more exposed to the environment. These findings are supported by the observation of a significant presence of scabs in the early days, in the wounds treated with *in natura *linseed oil or ESO. Since the two products occur in liquid form, they allow for faster absorption, however, increased exposure of the wound bed to the drying environment leads consequently to the formation of scabs. Demling and Desanti [26] reported that the use of dressings has several purposes, the first being the protection of the wound to minimize the chances of infection and loss of water and electrolytes, with consequent dehydration of the wound bed.

Bates-Jensen [27] reported that the presence of exudate in the first 48 to 72 hours after an incision is normal, however, after this period, any further exudate is a sign of damage to the healing process. When exudate persists, disintegration of the scab occurs which favors the growth of microorganisms between it and the granulation tissue [28]. In our study we observed exudate in the first 48 h in all groups, the absence of exudate could be related to the antimicrobial activity attributed to essential fatty acids. According to Declair, EFA has bactericidal action due to its pH, which interferes with the permeability of the bacterial membrane, in addition to the fact that linoleic acid, when used in venous ulcers, allowed the elimination of infection in the lesions [29].

In animals treated with SSFLO (1%, 5%, or 10%) we observed a significant amount of fibrin (slough) compared to the ESO control. According to Kentlloyd [30], low oxygen tension, stemming from the closure of wounds, promotes the accumulation of fibrin in the center of the lesion and stimulates the proliferation and centripetal migration of fibroblasts along the fibrin mesh. Thus, the difference was significant in the products that had petroleum jelly as a vehicle, which allowed for greater occlusion of the lesion with stimulation at low oxygen tension. Our results were similar to those of Otranto et al. [2], who evaluated the healing activity in rats and found a significant difference in the amount of fibrin (slough) in animals that were orally supplemented with linseed oil when compared with the control group, supplemented with water. The same authors also warned that the use of linseed and fish oil should be avoided whenever there is a tendency to fibrosis, as in hypertrophic scars and keloids.

The significantly earlier appearance of granulation tissue around of the wound in animals treated with the 1% or 5% SSFLO, when compared to the ESO control group, can probably be attributed to the semisolid formulation, which presents in its composition an emollient and occlusive vehicle (petroleum jelly), which allowed the wound to remain hydrated, due to the occlusion process that decreased the net loss to the environment [31].

Histological analysis performed at the end of the experiment (day 14) revealed that the groups treated with SSFLO (1% or 5%) showed significant reepithelialization compared to negative control (petroleum jelly), but the same was not observed in groups treated with 10% SSFLO or *in natura *linseed oil. One possible explanation for the absence of significant reepithilialization in these groups can be attributed to the presence of a high concentration of *ω*-3 PUFA in relation to the preparations of 1% and 5% SSFLO. According to Silva [31], the active compounds present in the formulations for dermatological use pass through the corneal stratum by passive diffusion. As this mode of transport is concentration-dependent, it would imply a greater dermal absorption of the PUFAs present in 10% SSFLO and in *in natura* linseed oil. Studies by Cardoso et al. [8] showed that high concentrations of *ω*-3 PUFA promote delayed healing of skin wounds in rats treated topically with this product. Additionally, Scardino et al. [32] also showed delayed wound healing as reflected in the decrease of reepithelialization of skin wounds in dogs fed a diet rich in *ω*-3 PUFA. According to Altavilla et al. [33] the delay in the healing process could be due to the presence of greater numbers of unsaturated bonds in *ω*-3 PUFA, which is predisposed to lipid oxidation and consequently a delay in wound healing.

In the present work, we have also observed a significant amount of inflammatory cells in the group treated with 10% SSFLO. These results corroborate those found by Otranto et al. [2], which demonstrated a significant amount of inflammatory cells in skin flaps of rats given a diet rich in linseed oil during the process of tissue repair. According to McDaniel et al. [34], people supplemented with a diet rich in *ω*-3 PUFA levels showed elevated proinflammatory cytokines in skin tissue. Studies have shown that PUFAs are main precursors of many lipid mediators involved in the inflammatory response, such as vascular contraction, chemotaxis, adhesion, transmigration and cell activation, and these are important functions of the inflammatory phase of tissue repair [35, 36].

We did not observe significant differences with respect to the density of collagen and skin resistance to mechanical traction in the groups treated with SSFLO (1%, 5%, or 10%) when compared to the ESO and petroleum jelly control groups. Our results are consistent with those of Albina et al. [37] who studied the influence of a diet rich in *ω*-3 PUFA in the deposition of collagen (after 30 days) and mechanical resistance of rat skin to traction (after ten days) of the incision, and who found no significant difference among groups.

**Figure 5:**
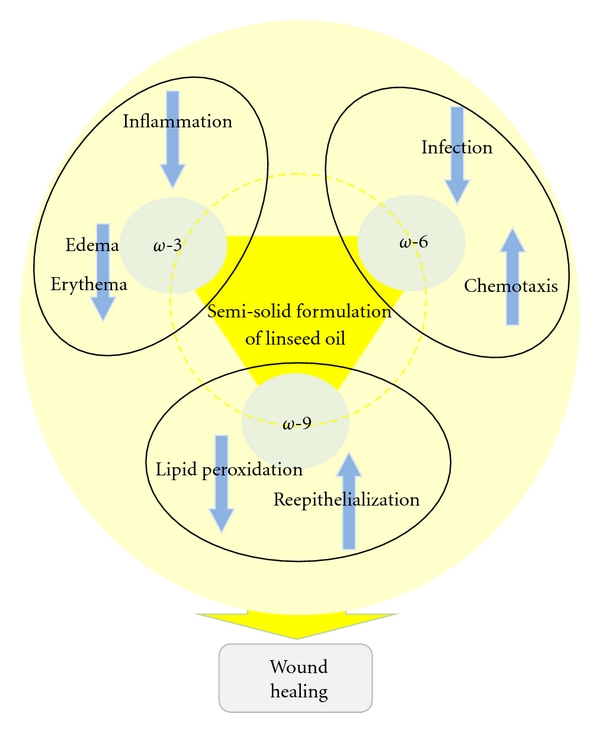
Possible role of the fatty acids linolenic acid (*ω*-3), linoleic acid (*ω*-6) and oleic acid (*ω*-9) present in semisolid formulation of linseed oil on wound repair.

Our study showed that topical administration of SSFLO (1% or 5%) in experimental excisional wounds promotes reepithelialization in 100% of the animals treated, therefore indicating the potential for therapeutic action of linseed oil (Figure 5), when used at low concentrations in the preparation of dermatological formulas with a solid base to be used in the process of dermatological reparations.
